# Roles of Identified Long Noncoding RNA in Diabetic Nephropathy

**DOI:** 10.1155/2019/5383010

**Published:** 2019-02-12

**Authors:** Yan Li, Keyang Xu, Kechen Xu, Sixiang Chen, Yifang Cao, Huakui Zhan

**Affiliations:** ^1^The First Clinical Medical College, Chengdu University of Traditional Chinese Medicine, Chengdu, 610075 Sichuan, China; ^2^Zhejiang Chinese Medical University, Hangzhou, 310053 Zhejiang, China; ^3^The Fourth Affiliated Hospital, Zhejiang University School of Medicine, Yiwu, 322000 Zhejiang, China; ^4^The First Hospital of Jiaxing, Jiaxing, 314001 Zhejiang, China

## Abstract

Diabetes mellitus is the leading chronic disease in the world, and diabetic nephropathy (DN) as one of its complications could increase the mortality. The development of DN is associated to abnormal hemodynamic factors like cytokine networks and the intervention of metabolic risk factors like blood pressure, blood glucose, and blood lipid. However, the pathogenesis of DN is still poorly understood. Although glucose-lowering drugs and insulins have significant effects on blood glucose, the fluctuation of blood glucose or other risk factors could continuously damage the kidney. Recent studies reported that the progression of DN is closely related to the expression of long noncoding RNA (lncRNA), which is important for the early diagnosis and targeted intervention of DN. In this review, we briefly summarize the published studies on the functions and potential mechanism of reported lncRNA in the regulation of DN.

## 1. Diabetic Nephropathy

Diabetic nephropathy (DN) is a progressive kidney disease that develops consequently to diabetes and is the important cause of chronic renal disease worldwide [1]. And DN accounts for approximately 40% of diagnosed end-stage kidney failure [2]. The early features of DN include glomerular mesangial expansion, hypertrophy, and increased renal accumulation of extracellular matrix (ECM) proteins such as collagens and fibronectin, as well as podocyte effacement [3, 4]. Albuminuria is used to stage DN and is regarded as a biomarker for diagnosis [5, 6]. But the typical pathological characteristics of DN can also be characterized by excessive proliferation of ECM and diffuse glomerular basement thickening of mesangial cells (MCs), which can eventually lead to glomerular sclerosis and renal interstitial fibrosis when exposed to high glucose [7, 8], because MCs can secrete various cytokines, such as transforming growth factor *β*1 (TGF-*β*1), collagen (COL), and fibronectin (FN) [9]. In addition, genetic factors are also important for disease risk [10, 11]; recent evidence has shown the involvement of epigenetic factors, such as DNA methylation, histone posttranslational modifications, microRNAs (miRNAs), and lncRNAs which are involved in the development of renal diseases, including DN [11]. It has been reported that many factors which are crucially linked with the progress of DN, such as inflammation, oxidative stress, activated hexose, renal ECM, and the polyol pathway, are related to epigenetic factors [5].

## 2. Long Noncoding RNA (lncRNA)

lncRNA was first described in the large-scale sequencing of full-length eDNA library in mice in 2002. lncRNA is a group of transcription materials with >200 nucleotides, which lacks a specific complete open reading frame and has no protein-coding function [12]. lncRNA accounts for 80% in the whole mammalian genome transcripts [12]. The majority of lncRNAs are produced by the transcribing of RNA polymerase [13]. Similar to mRNA, lncRNA is commonly expressed in eukaryotic genomes with 5′cap structures and a poly adenosine tail [14]. According to the location of lncRNA and proximal protein-coding genes in the genome, lncRNA can be classified into six categories: exon sense overlapping, intron sense overlapping, intronic antisense, natural antisense, and bidirectional and intergenic lncRNA [15]. Recent studies have shown that lncRNA regulates gene expression on a variety of levels, mainly including epigenetic transcription and posttranscriptional modification, and the regulation modes include chromosomal modification, transcriptional interference, or transcriptional activation [16]. Expression disorders of lncRNA are found in many types of tumors and neurological and cardiovascular diseases [17, 18]. Accumulating evidence has indicated the significant roles of lncRNAs in the pathophysiology of DN, and the crosstalk between lncRNA and DN was widely reported in recent years [19–21]. lncRNAs are involved in the procession of DN through regulating many important factors, such as pathologic process in MCs, reactive oxidative products (ROS), mechanisms involving ECM accumulation, and actions of miRNAs [1, 22, 23]. For example, ENSMUST00000147869 is downregulated in diabetic renal tissue, and mesangial cell proliferation and fibrosis are significantly enhanced through silencing its expression [24]. ROS-induced expression of ASncmtRNA-2 may contribute to DN fibrosis through regulating TGF-*β*1 [25]. CYP4B1-PS1-001 is significantly downregulated in early DN, and it can significantly inhibit the proliferation and fibrosis of MCs through inducing its overexpression [26]. NR_033515 promoted cell proliferation, fibrogenesis, and the EMT (epithelial-mesenchymal transition) process by miR-743b-5p [1]. Therefore, abnormal expression of lncRNA plays key roles in the occurrence and development of DN.

## 3. lncRNA Upregulated in DN

### 3.1. LncPVT1

PVT1 (plasmacytoma variant translocation 1) is a lncRNA (1.9 kb) that encodes a number of alternative transcripts. When it is amplified and overexpressed, it will increase cell proliferation and inhibit apoptosis [27]. PVTI is the first ncRNA reported to associate with kidney disease [28], which is highly expressed in human renal MCs under a high-glucose condition and significantly promotes the expression of fiber connection protein (FN1), type IV collagen, TGF-*β*1, and type 1 plasminogen activator inhibitor (PAI-1). More importantly, the deletion of PVT1 gene in MCs significantly reduced the expression of major ECM proteins and their regulatory factors, including FN1, COL4A1, TGF-*β*1, and PAI-1 [1, 29], while PAI-1 is the main inhibitor of glomerular ECM degradation [30]. It indicates that PVT1 may participate in the genesis and development of DN by regulating the accumulation of ECM. Some studies also suggest that some of the effects of PVT1 on ECM factors may be mediated through the actions of miRNAs, such as miR-1207-5p and miR-1207-3p [1].

### 3.2. lncRNA MALAT1

Metastasis-associated lung adenocarcinoma transcript 1 (MALAT1) is broadly expressed in mammalian tissues including the kidney and in tumors [31]. And MALAT1 is also aberrantly upregulated in early DN [32]. *β*-Catenin is a key mediator in the WNT signaling pathway, which can contribute to podocyte malfunction and albuminuria as well as kidney fibrosis [33]. MALAT1 could promote the translocation of *β*-catenin into the nuclei via enhancing serine/arginine splicing factor 1, and nuclear accumulation of *β*-catenin can cause podocyte damage and eventually lead to DN [20, 34, 35]. In addition, MALAT1 is a real culprit as an initiator of inflammation and oxidative stress, which can regulate glucose-induced upregulation of inflammatory mediators IL-6 and TNF-*α* through activation of serum amyloid antigen 3 (SAA3) [36]. Such changes may influence endothelial stability which is essential for all organs and for macro- and microvessels, which in the end leads to DN [37–39]. Furthermore, MALAT1 regulates renal tubular epithelial pyroptosis by modulated miR-23c targeting of ELAVL1 in DN [40]. Therefore, MALAT1 may be a potential therapeutic target for DN.

### 3.3. LincRNA Gm4419

Gm4419 (Ensembl ID ENSMUST00000180671) is a LincRNA, which is located in chromosome 12 (Chr12:21417911-21419803, 1730 bp) [41], and it is a regulator of the transcription factor nuclear factor kappa light-chain enhancer of activated B cells (NF-*κ*B), which is a crucial inflammatory stimulus for DN [42]. Gm4419 can directly interact with p50 to regulate the NF-*κ*B/NLRP3 inflammasome signaling pathway and mediate inflammatory molecular expressions in MCs, and it is associated with the development of inflammation, fibrosis, and proliferation of MCs with high glucose [23]. The silencing of Gm4419 expression leads to significant inhibition of cell inflammation, fibrosis, and proliferation in MCs with high-glucose conditions [23]. Thus, Gm4419 may have a functional role in DN inflammation through NF-*κ*B/NLRP3 inflammasome signaling and may act as a novel and specific therapeutic target for DN.

### 3.4. lncRNA GM5524

Cellular autophagy and apoptosis are essential for the maintenance of normal tissue homeostasis under physiological conditions [43]. The disorder of cellular autophagy and apoptosis has been reported in diabetes and its complications [44]. Gm5524 is significantly upregulated in DN tissues and podocytes under high-glucose conditions [45]. Gm5524 may be involved in DN by affecting these two processes: the first process is Gm5524 having effects on apoptosis and autophagy-associated factors through inhibiting antiapoptotic Bcl2 protein expression. And proapoptotic protein Bax expression is increased in Gm5524 knockdown podocytes [46, 47]. The second process is Gm5524 which promotes the development of DN by activating the LC3/ATG signaling pathway, which is a well-established biochemical assay to determine the activation of autophagy [45]. Thus, Gm5524 may further the understanding of the involvement of lncRNAs in DN.

### 3.5. lncRNA NR_033515

NR_033515 is significantly upregulated in serum of DN patients, and the expression level is related to the different stages of DN and positively associated with diagnostic markers of DN (KIM-1 and NGAL). Overexpression of NR_033515 promotes MC proliferation and inhibits MC apoptosis. And it also increases the expression of proliferation-related genes (PCNA and cyclin D1), fibrogenesis-related gene proteins (P38, ASK1, fibronectin, and *α*-SMA), and EMT biomarkers (E-cadherin and vimentin) by regulating miR-743b-5p expression [19]. Thus, NR_033515 may be a pivotal target for the early diagnosis and treatment of DN.

### 3.6. lncRNA Erbb4-IR

Erbb4-IR is located within the intron region between the first and second exons of ErBb4 gene on chromosome 1 of the mouse genome. A pathogenic role of Erbb4-IR is revealed in human T2DN tissues, and its molecular mechanism is also elucidated in a novel T2DN mouse stain in S3KO db/db mice [48]. The functional role of Erbb4-IR in T2DN is revealed by kidney-specific silencing of Erbb4-IR to protect against the development of T2DN such as elevated microalbuminuria, serum creatinine, and progressive renal fibrosis in db/db mice. In addition, Erbb4-IR can directly inhibit the transcription of renoprotective miR-29b, and then TGF-*β*/Smad3 signaling is activated. Therefore, renal fibrosis and renal dysfunction are largely promoted by Erbb4-IR during the progression of T2DN [49]. Thus, Erbb4-IR may represent a precise therapeutic target for DN.

### 3.7. lncRNA ASncmtRNA-2

Antisense mitochondrial noncoding RNA-2 (ASncmtRNA-2) is a mitochondrial lncRNA that is expressed in the mitochondria and exported to the nucleus [50, 51]. Previous studies have revealed that it is involved in the tumorigenesis and mitochondrial retrograde signaling pathways [51]. Notably, it has been demonstrated that ASncmtRNA-2 is overexpressed during aging and replicative senescence in human endothelial cells [52]. And ASncmtRNA-2 potentially serves a role in physiological oxidative stress and overproduction of oxidative products such as ROS, which can induce injury to the human kidneys through the following mechanisms: (i) inducing lipid peroxidation, protein crosslinking, and the formation of DNA adducts, leading to tissue damage; (ii) inducing direct damage to cellular DNA; and (iii) activating multiple cellular signaling pathways, including NF-*κ*B and TGF-*β*1. These mechanisms induce further generation of ROS, synthesis, secretion of cytokines, and deposition of ECM components, which induce more severe damage to the kidneys. Therefore, ASncmtRNA-2 can be a novel method to regulate ROS generation to reduce renal damage in DN in a clinical setting [25, 53–55].

### 3.8. lncRNA Lnc-MGC

Lnc-MGC can serve as a scaffold for a cluster of 40 miRNAs and appears to induce features of early DN [56]. Lnc-MGC can regulate megacluster (MGC), and its 3′ region overlaps with Mirg, and the middle region with Gm2922, other ncRNAs. Lnc-MGC is regulated by an endoplasmic reticulum (ER) stress-related transcription factor, CHOP (C/EBP homologous protein), via TGF-*β*1-dependent and TGF-*β*1-independent pathways [57]. ER stress has been observed to be increased in patients with progressive DN, and expression of renal CHOP and albuminuria is significantly increased in aged diabetic mice by promoting ER stress [58, 59]. Furthermore, a chemically modified oligonucleotide (gapmer) targeting Lnc-MGC can inhibit cluster miRNAs and then decrease protein synthesis, ER stress, glomerular ECM, and hypertrophy in diabetic mice and human DN tissues [57]. These results demonstrate the translational implications of targeting Lnc-MGC for controlling DN progression.

## 4. lncRNAs Downregulated in DN

### 4.1. lncRNA TUG1

lncRNA taurine-upregulated gene 1 (TUG1) was firstly identified as a part of photoreceptors and retinal development in mouse retinal cells, a lncRNA located at chromosome 22q12 [60]. TUG1 was considered to be involving in regulating carcinogenesis in several malignant tumors, and it also has been reported to play a key role in the progression of DN [61]. TUG1 is significantly repressed in the podocytes of diabetic mice by rescuing PPAR*γ* coactivator *α* (PGC-1*α*) expression, which is an important member of the nuclear receptor superfamily and well known to have an important role in mitochondrial bioenergetics and respiration [62, 63], and ameliorating the courses of DN, for regulating glomerular MCs proliferation, cell cycle, and diabetic glomerular ECM synthesis [64–66]. TUG1 acts as an endogenous sponge of miR-377 and downregulates miR-377 expression levels and thereby can relieve the inhibition of its target gene PGC-1*α* and alleviates ECM accumulation and cytokine secretion in MCs, including PAI-1, TGF-*β*1, FN, and collagen IV (Col IV) under high glucose [67]. Overall, TUG1 provides a novel insight of DN pathogenesis.

### 4.2. lncRNA MIAT

Myocardial infarction-associated transcript (MIAT), also known as retinal noncoding RNA 2 (RNCR2), is identified in myocardial infarction [68]. Dysfunction of kidney tubules in the tubular system of a diabetic kidney is proposed as the initial event in the development of DN [69]. In diabetic rats, MIAT shows the lower level and its expression is negatively correlated with serum creatinine and BUN [70]. MIAT can regulate proximal convoluted tubule cell viability via stabilizing nuclear factor erythroid 2-related factor 2 (Nrf2) expression, which is the key molecule of cellular defense against high blood glucose-induced oxidative stress and genotoxicity of cells. And Nrf2 can pathologically and functionally protect the kidney against diabetic damage [71, 72]. Interestingly, expression of Nrf2 can be enhanced by MIAT overexpression in 45 mM glucose-incubated renal tubular epithelial cell line (HK-2 cells) [70]. In summary, the data suggest that MIAT/Nrf2 served as an important signaling pathway for DN and it might be the potential therapeutic to reduce the burden of this disease.

### 4.3. lncRNA CASC2

Cancer susceptibility candidate 2 (CASC2) has been showed to have critical functions in tumorigenesis [73]. Recently, it was reported that the low expression of CASC2 has diagnostic values in serum and renal tissues for diabetes complicated with chronic renal failure [74]. A follow-up showed that patients with low serum level of CASC2 had significantly higher incidence of chronic renal failure by inhibiting the JNK pathway, which is common in patients with DN. Overexpression of CASC2 significantly inhibited the apoptosis of podocytes. In addition, treatment with a JNK activator significantly reduced the inhibitory effects of CASC2 overexpression on apoptosis of podocytes [75, 76]. Furthermore, the ROC curve analysis showed that the CASC2 level in renal tissues and serum is effective in diagnosing type 2 diabetes complicated with chronic renal failure [74]. Thus, CASC2 may serve as a predictive factor and target for the treatment and prevention of DN with chronic renal failure.

### 4.4. lncRNA ENSMUST00000147869

ENSMUST00000147869 has been found to be downregulated in DN, proliferation and fibrosis indexes are reversed in MCs with the action of Cyp4a12a, a neighboring gene locus to ENSMUST00000147869, and it is the predominant 20-hydroxyeicosatetraenoic acid synthase involved in determining sex- and strain-specific differences in susceptibility to hypertension and other cardiovascular diseases [77]. Cyp4a12a is a member of Cyp4a isoforms. CYP4 proteins metabolize fatty acids, eicosanoids, and vitamin D and are important for chemical defense, and the production of kidney CYP4 arachidonic acid metabolites can contribute to the abnormalities in renal function [78]. Downregulated Cyp4a12a is defined as a target gene, which can be recruited during ENSMUST00000147869 overexpression [77]. ENSMUST00000147869 can affect the synthesis of ECM and dramatically decreased the levels of fibronectin and Col IV in MCs under a high-glucose condition [24]. So the overexpression of ENSMUST00000147869 can significantly reduce the expression of the proliferation index (PCNA and cyclin D1) and fibrosis index (collagen I and FN) in MCs, as well as the growth rate. Thus, intergenic lncRNA ENSMUST00000147869 with nearby Cyp4a12a can be regarded as the potential therapeutic target and molecular biomarker for DN.

### 4.5. LincRNA 1700020I14Rik

1700020I14Rik is located in chromosome 2 (Chr2: 119594296–119600744) [79], which has been found to be downregulated and acts as an endogenous RNA to regulate the miRNAs in DN. The induced overexpression of 1700020I14Rik can reduce the expression of miR-34a-5p via the silent information regulator T1/hypoxia-inducible factor-1*α* (Sirt1/HIF-1*α*) signal pathway and eventually promotes proliferation and fibrosis in MCs [22]. Intriguingly, it has been reported that Sirt1 is a direct target of miR-34a-5p [22, 42], so the Sirt1/HIF-1*α* signaling pathway plays a significant role in the proliferation and fibrosis of DN [80]. However, knockdown of 1700020I14Rik will reverse the upper processes. Furthermore, the expressions of renal fibrosis genes including TGF-*β*1, FN, and Col IV also decreased by induced overexpression of 1700020I14Rik [22]. These results provide new insights into the regulation between 1700020I14Rik and miR-34a-5p/Sirt1/HIF-1*α* signaling pathway during the progression of DN.

### 4.6. lncRNA CYP4B1-PS1-001

CYP4B1-PS1-001 is located within a cluster of genes on chromosome 4 related to cytochrome P450 (CYP450) and is important in many reactions involving drug metabolism and synthesis of cholesterol, steroids, and other lipids [81]. CYP4B1-PS1-001 is significantly downregulated in response to early DN. While overexpression of CYP4B1-PS1-001 can inhibit proliferation and fibrosis of MCs due to an interaction with nucleolin (NCL). Furthermore, degradation of CYP4B1-PS1-001-associated NCL is mediated by a ubiquitin proteasome-dependent pathway [26]. The results show that overexpression of CYP4B1-PS1-001 decreases the levels of FN and collagen I as the major components of ECM in MCs under a high-glucose condition [81]. Overall, CYP4B1-PS1-001 could provide a potential therapeutic target and molecular biomarker in DN pathogenesis.

### 4.7. lncRNA Gm15645

Gm15645 is significantly downregulated in DN tissue podocytes in a high-glucose condition. The mechanism of Gm15645 is opposite with that of Gm5524, which may affect podocyte apoptosis and autophagy via regulation of the Bcl2/Bax and LC3/ATG pathways in DN [45].

### 4.8. lncRNA LINC01619

LINC01619 can regulate miR-27a/FoxO1 (forkhead box protein O1) and endoplasmic reticulum (ER) stress-mediated podocyte injury in DN by serving as a “sponge” for miR-27a. FOXO1 is the earliest discovered transcription factor of the FOXO subfamily and plays an important physiological function in proliferation, apoptosis, differentiation, oxidative stress, and other biological processes involved in cell metabolic diseases such as diabetes [82]. FOXO1 abolishment not only upregulates CHOP and GRP78 expression in podocytes but also increases podocyte foot process effacement [83]. Thus, the recovery of LINC01619 can alleviate oxidative stress and podocyte injury, and the silence of LINC01619 can induce oxidative stress and podocyte injury, diffuse podocyte foot process effacement, and decrease renal function [83]. Downregulation of LINC01619 contributes to proteinuria and declines renal function in DN patients; therefore, targeting LINC01619 may be a therapeutic approach for preventing DN.

## 5. Conclusion

**Figure 1:**
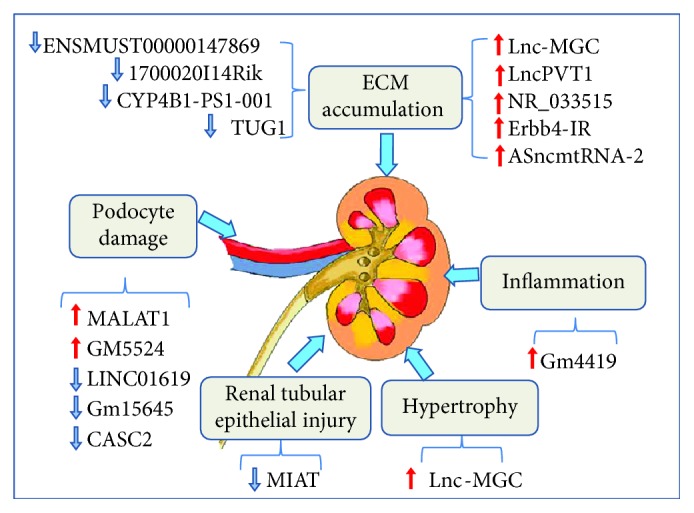
The roles of lncRNAs in diabetic nephropathy. Dysregulation of upregulated lncRNA (LncPVT1, MALAT1, Gm4419, GM5524, NR_033515, Erbb4-IR, ASncmtRNA-2, and Lnc-MGC) and downregulated lncRNA (TUG1, MIAT, CASC2, ENSMUST00000147869, 1700020I14Rik, CYP4B1-PS1-001, Gm15645, and LINC01619) involved in ECM maintenance and regulation of inflammation and podocyte damage, as described in the text. Red arrows indicate the damaging function of lncRNA upregulated in DN, and blue arrows indicate the protective functions of lncRNA downregulated in DN.

lncRNAs play a crucial role in the pathogenesis and progression of DN. The upregulated lncRNAs have a common function: they can promote the excessive proliferation of ECM, glomerular sclerosis and renal interstitial fibrosis, and inflammation and thickening of MCs. On the contrary, the downregulated lncRNAs appear to function as the protective factor against DN (Figure 1). In the previous studies, most studies of DN were focused on the functions of miRNAs. The regulation of lncRNA in DN is complex, involving interactions among multiple molecules and signaling pathways. Detailed functional verification in multiple models of DN is essential for the identification of lncRNA with clinical application potentials. Furthermore, recent studies suggested that N6-methyladenosine (m^6^A) modification of lncRNAs may play key roles in many diseases, such as cancer, leukaemia, Parkinson's disease, obesity, and diabetes [84, 85]. Regulatory roles between lncRNAs and m^6^A methylation in DN need to be further clarified.
